# Young Adult Carers in the UK—New Evidence from the UK Household Longitudinal Study

**DOI:** 10.3390/ijerph192114076

**Published:** 2022-10-28

**Authors:** Giorgio Di Gessa, Baowen Xue, Rebecca Lacey, Anne McMunn

**Affiliations:** Department of Epidemiology and Public Health, University College London, London WC1E 7HB, UK

**Keywords:** caregiving, care provision, young people, prevalence, duration, inequalities

## Abstract

Despite growing interest in young adult carers, little is known about trends in prevalence of caregiving among young adults aged 16–29. Furthermore, few studies have so far investigated demographic, health, and socioeconomic inequalities in the duration of care among young carers as well as demographic differences in caregiving characteristics. Using data from 11 waves of the nationally representative UK Household Longitudinal Study (2009–2021), we first estimated the prevalence of caregiving among 16–29 years-old adults at each wave. Results show that about 9% of those aged 16–29 provided care, and that this prevalence remained stable throughout the 2010s. Then, selecting respondents who participated for three waves of more, we assessed demographic, socioeconomic, and health characteristics associated with duration of care using ordinal regression models. Almost 52% of carers cared at two or more waves. Compared to non-carers, those who cared had more disadvantaged socioeconomic backgrounds, were from ethnic minorities and reported poorer health, particularly if they cared at two or more waves. Finally, focusing on carers, we tested differences by sex, age, and urbanicity in care relationships, intensity, and duration. Overall, women and those aged 25–29 cared for longer hours, for more people, and for more years than men and younger carers respectively. Put together, these findings provide an up-to-date description of young carers in the 2010s in the UK.

## 1. Introduction

Interest in young people (defined as those aged 16–29) with caregiving responsibilities has grown considerably over recent decades. Early adulthood is considered a critical stage for people’s development, with many having not yet solidified their life plans and choices about work, marriage and parenthood. A growing body of work suggests that having caregiving responsibilities at younger ages might indeed have a negative impact on a range of outcomes, such as participation in social and leisure activities, educational opportunities, employment and career development as well as physical and mental health [1,2,3,4,5]. However, many important gaps in our knowledge of young carers remain. First, we know little about what percentage of young people provide informal care and whether this has changed in recent times, most likely because of the lack of representative annual data. Second, as most of previous studies are cross-sectional, we know little about how long young people engage in caregiving activities and whether providing care for longer disproportionally falls on those young people with fewer socioeconomic resources. Finally, few studies have so far provided a detailed picture of caregiving characteristics (such as hours of care, number of and relationship with recipients of care, and age of onset of care) among younger carers. Our study aims to describe and better understand demographic and socioeconomic differences in care provision among young people in the UK.

Provision of care among younger adults is likely to be on the increase, owing to several socio-demographic factors. For instance, increased life expectancy means that it is common for children and young adults to grow up while their grandparents, and even great grandparents, are living and may require help [6,7,8]. Similarly, delayed childbearing [9] has resulted in that a growing number of young adults grow up with older parents who might need care themselves or might require help with their caregiving duties. Aging populations and other relevant socio-demographic changes (such as stronger labour market ties for mothers, higher levels of divorce and separation, and smaller family sizes) are likely to result in a growing number of older people in need of care who are increasingly likely to have to rely on the help and support of any of a shrinking pool of their immediate family members, including younger adults [10]. Finally, although trends are country-specific, a non-negligible proportion of families live in three- and skipped-generation households where care and support exchanges between the younger and older generations are facilitated by their joint living arrangements [11,12].

While socio-demographic circumstances suggest the of prevalence of young carers may be increasing, so far evidence based on large-scale surveys remain limited, with most of the published studies reporting on underaged carers or very specific age groups which overlook possible age differences across young adulthood [5,13,14]. For instance, Nagl-Cupal et al. [5] reported care (estimated at 4.5%) among Austrian children aged 10–14 whereas Leu and colleagues (2019) investigate care among Swiss children aged 10–15 (finding a prevalence of carers of 7.9%). Moreover, even fewer studies have looked at whether and to what extent the prevalence of young carers has changed over time [15,16]. For instance, the 2016 Australia Census found that 5.6% of those aged 15–24 years reported informal care, a slight increase from 5.0% in 2006. These few studies are based on census data from only two time-points, with statistics that might rely on parents’ disclosure of the caregiving role of their children which can underestimate care prevalence among young people [17,18]. Therefore, to date, no studies have provided an overview of the annual prevalence of caregiving among young people over several years using a survey which, by design, collects information on different aspects of participants’ lives directly from each participant.

## 2. Variations in Informal Care Provision

Based on the ‘Informal Care Model’ (ICM) [19], many studies have examined individual variation in informal care provision (although this model was originally designed to study the *onset* of informal care). This framework posits that provision of care is not a random process, and that factors such as gender-related expectations around care as well as competence or financial resources might play an important role in understanding why and how an individual provides care. For instance, poor health of the carer is likely to limit the provision of care [20]. Cost/benefit calculations including potential loss of income, cost of formal care, or health/well-being consequences derived from caregiving might also shape both the decision to provide care, as well as the intensity of care provided [21]. For instance, people in full-time employment and higher earners are less likely to provide care, and if they do they tend to take on less intensive caregiving responsibilities [22]. In line with these arguments, in this study we use the ICM to address two important lacunae: how socioeconomic, health, and demographic characteristics are associated with duration of young adult care, as well as with caregiving characteristics.

So far, little is known about the duration of caregiving among young people and the characteristics of those who provide care for longer. This is important because if providing care for more years falls disproportionately on those with fewer resources, this may exacerbate existing socioeconomic inequalities in early life. Moreover, several studies (mostly on middle-aged and older caregivers) suggest that the duration of caregiving episodes might have consequences for both employment and mental health and quality of life [2], particularly when informal caregivers feel trapped in this role [23,24]. For young people, caregiving for more years may be particularly problematic at a time when many young adults tend to make important transitions, from starting work to moving out of their parents’ place and in with a partner [25]. The length of time a young person provides care may also contribute to the normalisation of the caregiving role and to expectations that they will continue in that role [26]. To date, studies on young carers have been based on cross-sectional data and therefore could not, by design, account for how long respondents had provided care. However, cross-sectional studies suggest that demographic and socioeconomic differences between young carers and their peers not providing care exist. Generally, young women are more likely to be carers than young men, with a growing feminization of care as youth age [5,13,15,16]. This sex difference has been found consistently across different age groups [27,28,29], with theories explaining such persistent gender inequality ranging from reflecting traditional gender roles to (lack of) independent economic resources [30,31]. Furthermore, young people in lower income or single parent households, and those with culturally and linguistically diverse backgrounds have been shown to be more likely to take on a caregiving role [14,18,32,33]. In line with these cross-sectional findings and following the ICM framework, we expect that providing care for longer falls disproportionally on those young people with fewer socioeconomic resources as they might be less likely to access, purchase, and use alternative forms of care, help, and support from the market.

While the ICM highlights the social and care context as well as characteristics influencing the probability of taking up care, few studies have investigated the amount and type of care provided by younger adults and how they differ according to carers’ demographic characteristics [16,33]. In particular, evidence consistently shows that men are less involved in care provision than women, providing generally fewer hours of care [34]. However, little is known about whether other caregiving characteristics such as the number of people cared for, care recipient, and duration of care also differ by carer’s sex, particularly among young adult carers. Similarly, despite age being an important factor that could not only influence young people’s ability to provide care, but also their level of commitment (such as number of hour of caregiving) and who they care for (depending on their transitions to both employment and parenthood), to our knowledge only Stamatopoulos [16] has, so far, provided age-differentiated patterns of caregiving among young carers, with carers aged 20–24 providing most senior care compared to younger carers. Finally, many studies on older informal caregivers have suggested rural-urban disparities in their caregiving responsibilities as often those residing in rural areas have reduced access to long-term care, professional services, and general income [35]. Even among young carers there are indications that the readiness of formal support via the community can influence individuals’ care uptake, with urbanicity often described as a proxy of availability and accessibility of formal care services and support [33] as well as of potential stigma and lack of privacy [36]. However, to our knowledge, no previous studies have investigated rural-urban differences at a population level in caregiving characteristics among young people.

## 3. Materials and Methods

### 3.1. Study Population

We based our study on the UKHLS [37], an ongoing nationally representative longitudinal household panel study, based on a clustered-stratified probability sample of UK households, with all adults aged 16+ in chosen households surveyed annually since 2009 and supplemented by specific additional samples added at subsequent waves (initial response rate of 57.3%). More details of the survey’s sampling frame, methodology, and questionnaires have been reported elsewhere [URL https://www.understandingsociety.ac.uk/ (accessed on 15 October 2022)]. Although different age ranges have been used to identify carers among ‘young adults’ [3,38,39,40], in this manuscript young people are defined as being between 16 and 29 years old in line with definitions used by EUROSTAT [URL https://ec.europa.eu/eurostat/web/youth (accessed on 15 October 2022)].

In order to provide the annual prevalence of informal care among young adults aged 16–29 in the 2010s, data were drawn from the first 11 waves of the study, collected between 2009 and 2021 and analyses were restricted to respondents aged 16–29 at each wave (with figures ranging from 11,526 at Wave 1 to 5727 at Wave 11). Exploiting the longitudinal nature of the dataset, we then assess socioeconomic differences by duration of care. In this case, we pooled samples across the first ten waves only, because wave 11 overlapped with the COVID-19 pandemic and both methods of collection and questions on caregiving (but also employment) changed compared to previous waves. Moreover, we selected any respondents aged 16–29 when first observed in any of the ten waves under study and who participated in at least three waves (N = 15,754 respondents, with mean and median number of waves equal to six, and about two thirds of respondents with 4 or more consecutive waves). This allows all participants to be observed for a similar length of time (regardless of their initial caregiving status) and to have the same ‘risk’ of care for one or more waves (our multivariable model outcome). After selecting respondents with complete information on all characteristics, our analytical sample consisted of 14,462 young adults. Finally, in order to describe caregiving characteristics and test whether they differ by gender, age, and urbanicity, we focused on carers in our longitudinal sample (N = 3185). Ethical approval for the UKHLS was obtained by the University of Essex Ethics Committee.

### 3.2. Measures of Caregiving

The variable for caregiving responsibilities was derived from two questions asked of respondents at each wave: ‘Is there anyone living with you who is sick, disabled or elderly whom you look after or give special help to (for example, a sick, disabled or elderly relative/husband/wife/friend, etc.)?’ and ‘Do you provide some regular service or help for any sick, disabled or elderly person not living with you?’. In our multivariate analyses, we distinguished between those who never cared, those who cared only once, and those who cared at two or more waves. We did not consider further classifications of duration of care as the vast majority of carers (70%) provided care only for one or exactly two waves.

Those who reported caregiving were then asked a series of questions on the total number of people they cared for (1, 2, or 3 and more); their relationship to each care recipient (including parent, grandparent, partner, sibling child, other relative, other non-relative); and the numbers of hours spent caregiving every week (where the 7-point scale response ranging from 0–4 to 100+ hours per week was re-categorised into 0–4; 5–9; 10–19; 20–34; 35+ h/week due to small cell counts at the upper extremes). For respondents who cared at 2 or more waves, we considered the (rounded) average number of people cared for across waves; their averaged weekly hours spent providing care across waves; and any recipient cared for throughout the study. Robustness checks that considered the highest values of number of care recipients and care intensity yielded similar results (available upon request).

### 3.3. Control Variables

In our multivariable analyses we controlled for a number of socioeconomic and demographic characteristics. In particular, we controlled for sex and age groups (16–17; 18–24; 25–29). These age groups were chosen to reflect the widely used groups that in scholarly literature distinguish between underaged young people (mostly in education and still legally ‘children’), young adults aged between 18 and 24 (who have the legal status of ‘adults’, and are mostly in full-time work), and those aged 25–29 (a life stage when the majority of people move in with their partners and have their first child) [25]. Ethnicity is grouped into White; Black; Indian; Pakistani or Bangladeshi; and other Asian/other ethnic groups. For marital status, we distinguished between respondents who were legally married, those who were co-habiting, or single. Urbanicity was dichotomised as urban or rural based on population size/density of where the respondent lives. To capture respondents’ socioeconomic characteristics, we controlled for household income, employment status, and self-reports of parents’ highest occupational class (measured using the National Statistics Socio Economic Classification—NSSEC) when the respondent was aged 14. In particular, we used quintiles of household income (measured by monthly total household net income divided by the OECD equivalence scale); for respondents’ occupational class, we distinguished between those not in employment, and those employed in managerial/professional, intermediate, and routine/manual jobs; and for parental occupation, we additionally accounted for those whose parents were not in the household when the respondent was aged 14. Finally, as measures of health, we considered self-rated health and longstanding illness or disability. Self-rated health (SRH) was measured using responses to a generic question (“In general, would you say your health is …”) on a 5-point ordinal scale (excellent, very good, good, fair, or poor); ‘fair or poor’ were grouped together as less than 2% reported poor health. Finally, individuals were classified as having disability if they reported any longstanding physical or mental impairment, illness or disability (without specifying the issue). All covariates were time-invariant and were measured for all respondents (irrespective of whether they were providing care or not) when the respondent aged 16–29 was first observed in the study; this approach allows us to be consistent for both caregivers and non-caregivers.

### 3.4. Statistical Analysis

First, we provide the prevalence of care among young adults aged 16–29 at waves 1 to 11, showing also the distribution of care by place of caregiving (inside the household, outside the household, or both) and by age groups (16–17; 18–24; 25–29). Second, using pooled data of waves 1–10, we present unadjusted and covariate-adjusted associations between socioeconomic, demographic, and health characteristics and duration of care (no care, cared once, cared at two or more waves). Covariate-adjusted models are obtained from ordinal logistic regression models. The proportionality of the odds for all covariates was examined using the Brant test, and it was relaxed for those variables violating this assumption. Finally, focusing on the longitudinal sample of carers, we showed differences in the nature and extent of caregiving by sex, age groups (16–17; 18–24; and 25–29), and urbanicity. All analyses were weighted to account for non-random participation at the interview and took account of the complex study design of UKHLS. We used a complete case analysis as the percentage of item missingness is less than 5%. All analyses were performed using Stata 16.

## 4. Results

### 4.1. Trends of the Prevalence of Care among Young Adults Aged 16–29—Descriptive Findings

**Figure 1:**
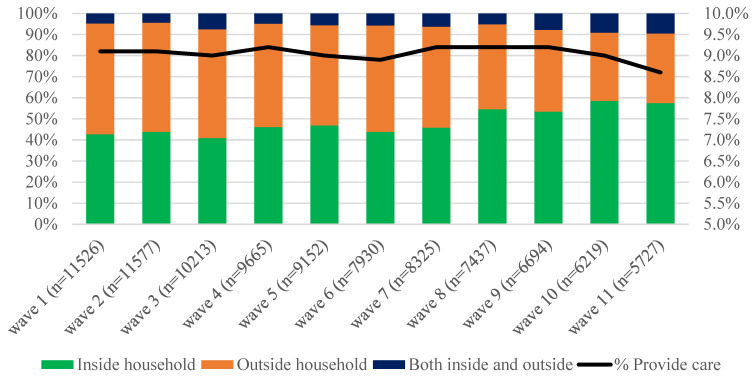
Prevalence of care among people aged 16–29 and distribution by place of care—UKHLS waves 1–11. Source: UK Household Longitudinal Study (UKHLS), Waves 1–11. Weighted data. Samples restricted to respondents aged 16–29 at each wave. Notes: the values on the right refer to the prevalence of care whereas those on the left to the distribution by place of care.

**Figure 2:**
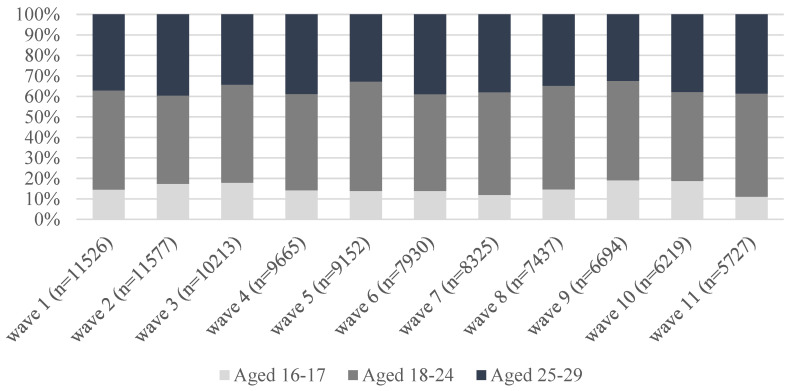
Distribution of care among people aged 16–29 by age groups—UKHLS waves 1–11. Source: UKHLS, Waves 1–11. Weighted data. Samples restricted to respondents aged 16–29 at each wave.

Figure 1 shows the prevalence of care among respondents aged 16–29 in the UK between 2009/10 (wave 1) and 2020/21 (wave 11). Overall, among respondents aged 16–29, the percentage reporting care provision was stable at ~9%. However, the distribution of place of care changed over the decade under study, with an increase in the percentage of both carers providing help inside of the household (from 43% in wave 1 to almost 59% in Waves 10 and 11) and those caregiving both in and outside the household (from less than 5% to more than 9%). Figure 2 shows the distribution of care by three broad age groups (16–17; 18–24; and 25–29): although we observe some fluctuations in the age distribution of carers, there are no clear patterns and the majority of young people who cared were aged 18–24 (between 43 and 53%) in all waves under study.

### 4.2. Associations between Demographic, Socioeconomic, Health Factors and Duration of Care

Table 1 shows the demographic, socioeconomic, and health characteristics of the sample by duration of care. Respondents who cared only once were, when they were first observed in the study, almost one year younger on average than those who never cared (20 years old vs. 20.8). Carers were more likely to be female, especially among those who cared for 2 or more waves. We also observed differences by ethnicity, with Pakistani/Bangladeshi respondents being more likely to provide care at two or more waves. Compared to non-carers, carers who cared once were more likely to be single, whereas those who cared for two or more waves to live in urban areas. When socioeconomic characteristics of both respondents and their parents were considered, results suggest that overall those who provided care were more likely to be in more disadvantaged socioeconomic positions, particularly if they reported care for two or more years. For instance, carers at two or more waves were less likely to be in professional or managerial occupations and more likely to be in the lowest income quintiles. Respondents who cared for two or more waves were also more likely to report fair or poor self-rated health and long-standing illnesses than those who never reported care.

Table 2 shows the associations between respondents’ characteristics and duration of care obtained from a fully adjusted ordinal logistic model. Most of the associations described in Table 1 hold also in the mutually adjusted model. For instance, women, Pakistani/Bangladeshi ethnic groups, those in the lowest income quintiles, in routine/manual occupations, as well as in poorer health were more likely to report care provision at more waves.

### 4.3. Caregiving Characteristics by Sex, Age Groups, and Urbanicity

Table 3 shows the caregiving characteristics among young adult carers and tests the differences by sex, age groups, and urbanicity. Overall, about 50% of carers spent 0–4 hs/w providing care (the lowest category in the questionnaire); the majority (92%) cared for only one person; the most frequently reported recipients of care are parents (42%) or grandparents (40%). Nearly half the carers (49%) reported this activity for only 1 wave, with almost 30% caregiving for 3 or more years. The age when respondents first reported care is roughly evenly distributed, with a slightly higher percentage of carers being first observed at ages 16–17 (that is the age when UKLHS respondents are first asked questions on caregiving). However, there were differences in the caregiving characteristics, mostly by sex and age. Female carers were more likely to report caregiving for longer hours, for more people, and for more years than male carers. Furthermore, they were more likely to care for siblings, children, and other relatives than male carers. When we considered age, we found an incremental engagement in care as youth aged, with women aged 25–29 more likely to care for longer hours than those aged 16/24. Furthermore, older carers aged 25–29 were more likely to care for their partners and children but less for grandparents and siblings than younger carers. No differences were found between carers in urban and rural settings, except that the latter were more likely to report fewer hours of care and to care for non-relatives.

## 5. Discussion

Although a non-negligible percentage of young adults engage in caregiving responsibilities, there remain important lacunae about trends in prevalence of care, socioeconomic inequalities in the duration of care, and demographic differences in caregiving characteristics. Using data from UKHLS, our aim was to provide a detailed description of these issues among young carers in the UK.

Despite growing concerns that provision of care among younger adults is likely to increase, we found little variation between 2009 and 2020 in the overall prevalence of care among UK people aged 16–29, with ~9% reporting provision of care. This stability of prevalence of young carers is in line with those studies that analysed trends in Australia, Canada, and the UK using census data [15,16]. The percentage of young carers observed in our study, however, is higher than that observed using 2011 Census Data (5.4% in England and Wales) and this could partly reflect differences due to the wording of the questions, suggesting a potential undercount of carers in the census, particularly if caregiving roles were reported by parents and not young people themselves. Overall, we also found that over time most young carers (about two thirds) provided care for someone inside their household and this could partly reflect the increase mean age at which young people move out of their parents’ home [25].

Exploiting the longitudinal nature of the dataset, we found that more than half of those who cared (51%) reported this activity at two or more waves, with 16% caregiving for more than four. When we analysed characteristics associated with duration of care, we found marked socioeconomic differences as the ICM would lead us to expect, with those financially worse off (lowest income quintiles, in routine/manual occupations, and from more disadvantaged families) more likely to provide care, and to care for longer. Adding to the existing cross-sectional knowledge suggesting inequality by caregiving status [14,18,32,33], our descriptive and multivariate results suggest that the provision of care for more than one wave falls disproportionally on women, those with a Pakistan/Bangladeshi background, and poorer health. Overall, these findings reinforce the idea that informal care, even at younger ages, is not evenly distributed across different socioeconomic and demographic groups, as hypothesised by the ICM. It is plausible that those with fewer economic resources are less likely to be able to access and purchase alternatives to family care and support. Moreover, the act of caring might itself be incompatible with employment or with more routine/manual occupations, particularly when providing care long hours or regularly. Finally, young carers might fall into poorer financial circumstances because the person they care for can no longer contribute to the household finances. In our study, young carers who care for a parent (41%) or for a partner (5%) are at increased risk of living in households that might be dependent upon a single income and/or benefits, as found in other studies [41,42]. Although disentangling the directionality of caregiving duration and socioeconomic disadvantage was beyond the scope of this paper, our analyses provide further evidence that young carers who cared for longer are also more likely to have fewer socioeconomic resources. This may exacerbate existing inequalities in early life at a time when many young adults make important transitions in their lives, from starting work to moving out of their parents’ place and in with a partner.

Furthermore, we found sex and age differences in the caregiving experience, with female carers and those aged 25–29 giving help for longer hours, for more people, and more years than male carers and those aged 24 or younger respectively. Studies on carers in mid and later life also show that women are more likely to be main carers; to provide more hours of care; and to carry out more domestic and personal tasks than men [34,43]. Our findings, therefore, suggest that this gendered experience of care provision is apparent already from younger ages, with a growing feminisation of care and possibly greater expectations of care placed on girls and as young carers get older [16,44]. Finally, we found that carers aged 25–29 were more likely to care for partners and children while younger ones for grandparents and siblings. This is in line with Stamatopoulos [16], who also found that age related to the type of care with younger carers (aged 15–17) mostly providing childcare, and carers aged 20–24 providing ‘senior’ care. This is likely to reflect different stages of life, as people in their late 20s are less likely to have a grandparent alive that those in late teens or early 20s [6,45], as well as more likely to be a parent and to have moved out of their family home [25]. Finally, we found very few urban–rural differences in caregiving characteristics: however, young carers in rural settings are more likely to provide care for friends and neighbours, and to provide fewer hours of care compared to those who live in cities, in line with studies suggesting that people in rural areas have stronger community relations than those in urban areas, and that are more likely to help non-family members.

### Strengths and Limitations

This study draws strength from using UK nationally representative surveys that have collected yearly information on caregiving for anyone aged 16 and older in the 2010s. To our knowledge, this was the first study to investigate annual prevalence of care among young people using a large scale nationally representative survey, and to investigate socioeconomic and demographic differences in duration of care and caregiving characteristics. Our contribution, however, should be considered in light of several limitations. For instance, UKHLS does not collect information on the reasons why people provide care, and information on caregiving activities and responsibilities (including personal care, general companionship, or practical help) is not asked consistently. Future studies, both quantitative and qualitative, are encouraged to investigate these aspects of care, and how they relate to socioeconomic and demographic factors. Moreover, although our data come from a large nationally representative sample of young adults in the UK, it is worth noting that our longitudinal study sample might be skewed towards the more socioeconomic advantaged (in line with the widely recognised effect that retention in cohort studies is higher among those who are more advantaged). It is therefore likely that our study underestimates the associations between socioeconomic factors and duration of care. Furthermore, it is possible that those who cared for two or more waves did so on separate spells. Although, in our study, 56% of respondents caregiving at exactly two waves reported care at two consecutive waves, further studies could investigate caregiving trajectories (to account for separate spells of care and for those who move in and out of this role) and how they relate to demographic and socioeconomic characteristics. Finally, although we acknowledge that the associations found in our study may also depend on the availabilities of formal care services and support for carers, no such data was available. Future studies are encouraged to explore this aspect, as well as the role of family-norms and employment policies, ideally comparing different countries.

## 6. Conclusions

Our work contributes to an emerging body of evidence on young adults who provide care in the UK. This robust demographic descriptive data is particularly important for young carers as between the ages of 16 and 29 (the focus of this study) individuals experience many important, arguably life-defining, transitions such as entering higher education, starting employment, and/or leaving home. Because of their caregiving responsibilities, a considerable number of young people might experience difficulties with many of these important transitions [2,44,46], with detrimental economic and health effects that might persist into later life. Given that the experience of care provision is not similar across young people of different socioeconomic and demographic background, policymakers should provide appropriate support and formal care services particularly to those young people with caregiving responsibilities from disadvantaged backgrounds, in an attempt to reduce inequalities in the distribution of family care. Furthermore, future research should aim to investigate how caregiving interacts with socioeconomic status to affect young people’ health and well-being and employment opportunities.

## Figures and Tables

**Table 1:** Percentage distribution of demographic, socioeconomic, and health characteristics by duration of care.

	Non-Carer (N = 11277)	Cared Once (N = 1573)	Care for 2+ Waves (N = 1612)	*p* Value
Age when first observed				
16–17	37.8	40.5	35.3	<0.001
18–24	35.1	42.2	43.6	
25–29	27.1	17.3	21.2	
Mean	20.84	20.05	20.56	<0.001
Sex				
Male	52.9	50.0	42.4	<0.001
Female	47.1	50.0	57.6	
Ethnicity				
White	89.0	86.8	86.9	<0.001
Black	3.3	3.9	3.0	
Indian	2.3	3.3	2.3	
Pakistani/Bangladeshi	2.4	3.7	5.8	
Other Asian	2.9	2.3	2.0	
Marital status				
Married	8.4	6.1	7.8	<0.001
Cohabiting	15.5	10.9	14.3	
Single	76.2	83.0	78.0	
Place of Living				
Rural Area	21.4	20.4	16.7	0.011
Urban Area	78.6	79.6	83.3	
HH income quintiles				
1 (lowest)	16.8	23.8	26.6	<0.001
2	19.1	24.1	24.3	
3	21.5	18.0	23.4	
4	23.5	19.4	16.2	
5 (highest)	19.2	14.7	9.6	
Occupation class				
Management/Professional	14.9	9.5	8.6	<0.001
Intermediate	9.4	7.5	7.1	
Routine/manual	19.0	20.6	20.9	
Not working	56.7	62.4	63.5	
Parental occupational class (at age 14)				
Management/Professional	44.2	36.9	25.4	<0.001
Intermediate	22.5	20.4	19.3	
Routine/manual	24.0	25.6	29.9	
Not working	8.8	16.5	25.1	
Not in household	0.5	0.6	0.4	
Self-reported health				
Excellent	25.1	21.3	19.3	<0.001
Very good	41.1	38.8	34.1	
Good	25.2	27.9	32.8	
Fair/poor	8.6	12.0	13.8	
Physical health				
No long-standing illness	85.0	80.8	74.6	<0.001
With long-standing illness	15.0	19.2	25.4	

Source: UK Household Longitudinal Study, Waves 1–10. Notes: Socioeconomic and health characteristics refer to the time point when respondents aged 16–29 were first observed in the study. Analysis based on respondents with no missing data on any variables (N = 14,462). All analyses are weighted. *p*-value from bivariate associations.

**Table 2:** Association between demographic, socioeconomic and health characteristics and duration of care (no care; cared once; cared for two or more waves). Odds Ratios and 95% CIs from covariate-adjusted generalised ordinal logistic model (with partial proportional odds).

	OR	*p*-Value	95% CI	
Age: 16–17	Ref			
18–24	1.14	0.078	0.99	1.31
25–29	0.70	0.001	0.57	0.87
Sex: Male	Ref			
Female	1.24 ^i^	<0.001	1.10	1.38
	1.42 ^ii^	<0.001	1.22	1.64
Ethnicity: White	Ref			
Black	0.89	0.368	0.68	1.15
Indian	1.30	0.121	0.93	1.81
Pakistani/Bangladeshi	1.46	0.011	1.09	1.97
Other Asian	0.74	0.093	0.52	1.05
Marital status: Married	Ref			
Cohabiting	0.95	0.670	0.73	1.22
Single	1.08	0.513	0.86	1.36
Place of Living: Rural Area	Ref			
Urban Area	0.92	0.305	0.78	1.08
Household income quintiles				
1 (lowest)	1.56	<0.001	1.23	1.98
2	1.55	<0.001	1.24	1.93
3	1.29	0.028	1.03	1.61
4	1.11	0.349	0.89	1.38
5 (highest)	Ref			
Occupation class				
Management/Professional	Ref			
Intermediate	1.08	0.611	0.81	1.43
Routine/manual	1.36	0.013	1.07	1.73
Not working	1.22	0.106	0.96	1.55
Parental occupational class (at age 14)				
Management/Professional	Ref			
Intermediate	1.15 ^i^	0.111	0.97	1.37
	1.38 ^ii^	0.005	1.10	1.72
Routine/manual	1.40 ^i^	<0.001	1.20	1.65
	1.81 ^ii^	<0.001	1.46	2.24
Not working	2.68 ^i^	<0.001	2.18	3.29
	3.42 ^ii^	<0.001	2.67	4.37
Not in household	1.14 ^i^	0.724	0.56	2.30
	1.00 ^ii^	0.997	0.42	2.38
Self-reported health: Excellent	Ref			
Very good	1.03	0.742	0.87	1.21
Good	1.28	0.004	1.08	1.52
Fair/poor	1.33	0.013	1.06	1.67
Physical health:				
No long-standing illness	Ref			
With long-standing illness	1.46	<0.001	1.26	1.69

Source: UK Household Longitudinal Study, Pooled data from Waves 1–10. Notes: Socio-economic and health characteristics refer to the time point when respondents aged 16–29 were first observed in the survey. For variables that violate the proportional odds assumption: (^i^) Coefficient for ‘any’ care compared to no care at all; (^ii^) Coefficient for care provided for two or more waves compared to any other responses (i.e., no care or care for only 1 wave). Weighted analysis.

**Table 3:** Percent caregiving characteristics among carers by sex, age groups, and urbanicity.

	Carers n = 3185	Male n = 1332	Female n = 1853	*p* Value	16/17 n = 1246	18/24 n = 1333	25/29 n = 606	*p* Value	Urban n = 2668	Rural n = 517	*p* Value
Weekly hours											
0–4	50.2	57.1	44.2	<0.001	55.5	48.2	44.0	<0.001	48.4	57.8	0.026
5–9	20.0	18.7	21.1		20.4	20.9	17.3		20.3	19.0	
10 to 19	14.4	13	15.7		13.8	14.4	15.6		14.8	12.6	
20–34	7.2	6.1	8.1		5.0	7.6	10.6		7.6	5.4	
35 or more	8.2	5.1	10.8		5.2	8.8	12.6		8.8	5.2	
N of people											
1	91.6	93.1	90.4	0.087	91.9	93.3	87.3	0.002	91.7	91.3	0.231
2	7.2	6.2	8.1		6.3	5.9	12.1		7.3	6.8	
3 or more	1.1	0.7	1.5		1.8	0.8	0.6		0.9	1.9	
Years of care											
1	49.2	53.3	45.6	0.003	52.6	48.3	44.2	0.169	48.0	54.2	0.232
2	21.5	22.4	20.8		22.1	21.3	20.8		22.0	19.5	
3	12.9	10.4	14.9		11.8	13	14.7		13.3	10.9	
4 or more	16.4	13.9	18.6		13.4	17.4	20.3		16.7	15.4	
Age care onset											
16/17	21.2	22.6	20.1	0.406	56.1	0.0	0.0	<0.001	20.2	25.9	0.240
18/19	13.6	14	13.2		22.6	11.7	0.0		13.4	14.2	
20/21	13.2	13.8	12.6		13.5	18.7	0.0		13.7	10.9	
22/23	11.8	11.4	12.3		5.3	22.9	0.0		11.8	12.1	
24/25	13.5	14.2	12.8		2.3	24.2	11.4		13.7	12.2	
26/27	12.4	11.9	12.7		0.1	13.5	34.0		13.1	9.2	
28/29	14.4	12.1	16.3		0.0	9.0	54.6		14.1	15.5	
Care recipient											
Parent	41.5	42.2	40.8	0.588	40.2	40.3	46.6	0.229	42.5	37.0	0.147
Grandparent	40.5	41.3	39.8	0.559	43.3	40.7	34.3	0.09	39.7	43.7	0.279
Partner	4.7	4.4	4.9	0.636	1.3	5.8	8.6	<0.001	4.6	4.7	0.955
Sibling	6.6	7.7	5.8	0.056	12.6	3.8	1.3	<0.001	7.0	5.2	0.207
Child	5.5	2.9	7.6	<0.001	0.8	7.0	11.2	<0.001	5.5	5.3	0.896
Other kin	9.8	7.4	11.8	0.001	7.8	8.7	16.0	<0.001	10.1	8.3	0.373
Non-relative	15.9	16.0	15.9	0.991	16.3	15.4	16.5	0.258	14.8	20.9	0.005

Source: UK Household Longitudinal Study. Pooled data from Waves 1–10. Note: analyses restricted to respondents who reported care. Weighted analysis. p-value from bivariate association.

## Data Availability

Understanding Society (UKHLS) data are available through the UK Data Service at URL https://beta.ukdataservice.ac.uk/datacatalogue/studies/study?id=6931 (accessed 6 September 2021).
